# Supplementary figures and images for: Mapping QTL for Phenological and Grain-Related Traits in a Mapping Population Derived from High-Zinc-Biofortified Wheat
Source: Plants (Basel). 2023 Jan 3;12(1):220. doi: 10.3390/plants12010220 (PMC9823887; doi:10.3390/plants12010220)

Supplementary Figure S1: Frequency distribution of DH, DM, PH, TKW, TW during 2017 – 18 (Y1), 2018 – 19 (Y2), 2019 – 20 (Y3), and across years.

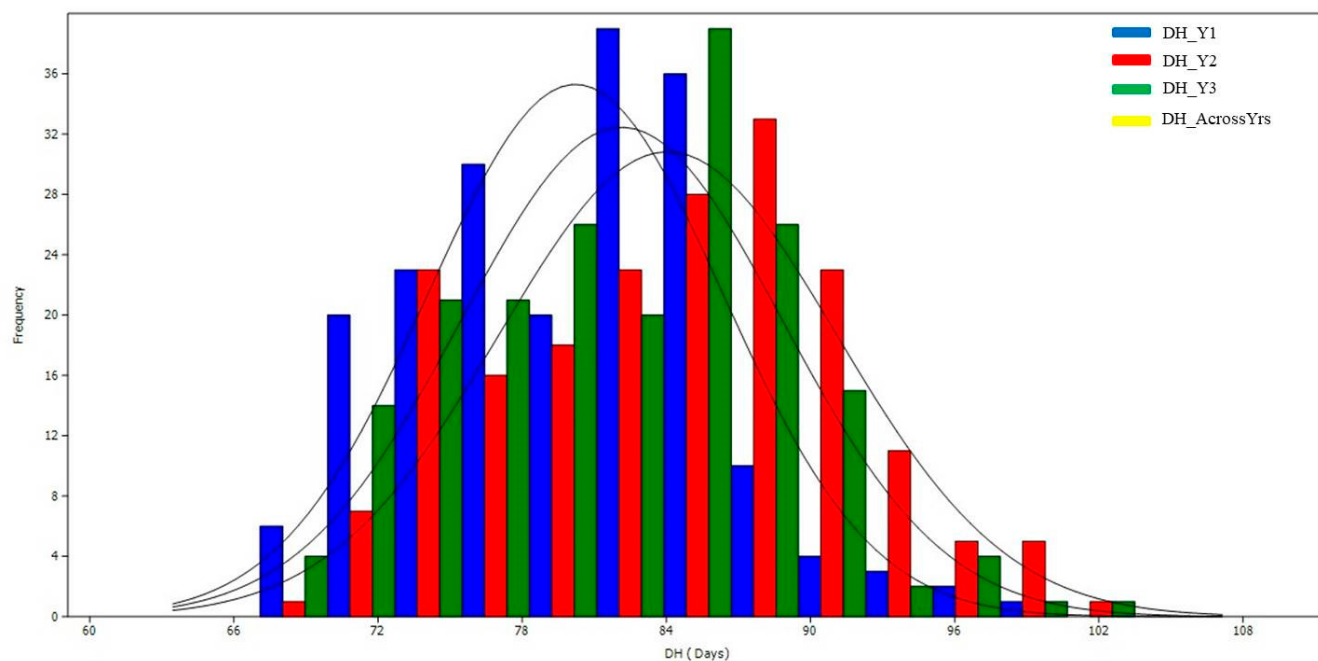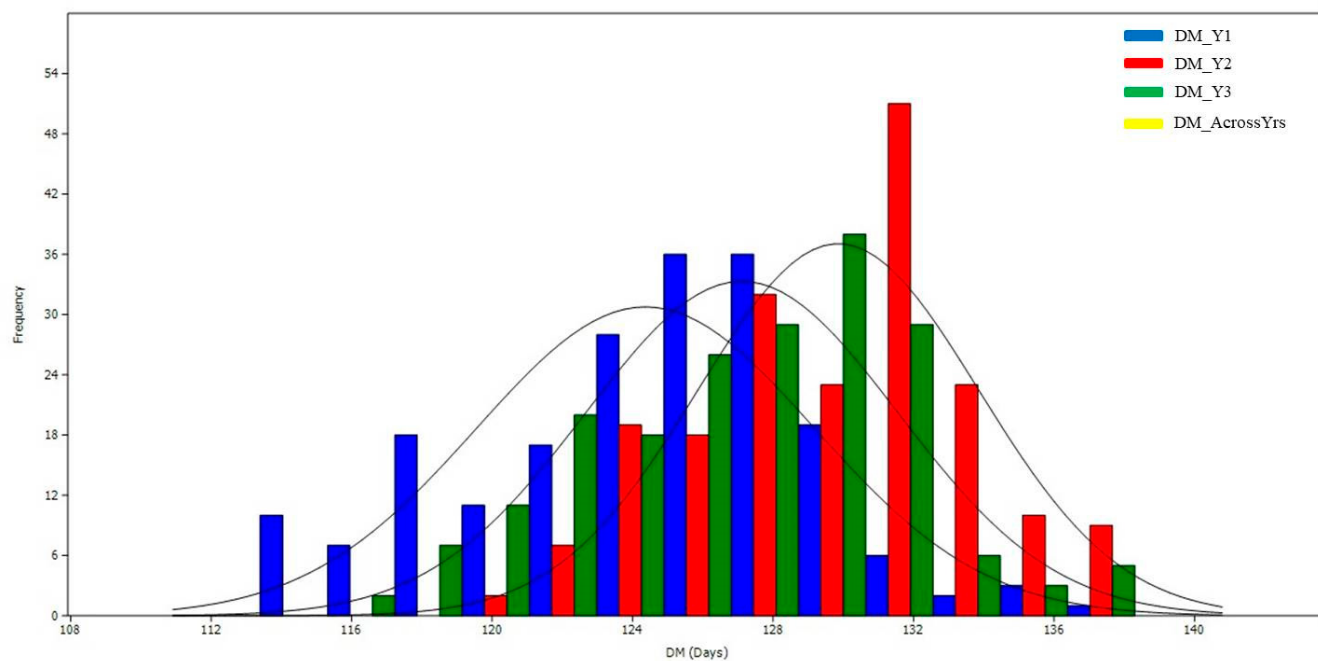

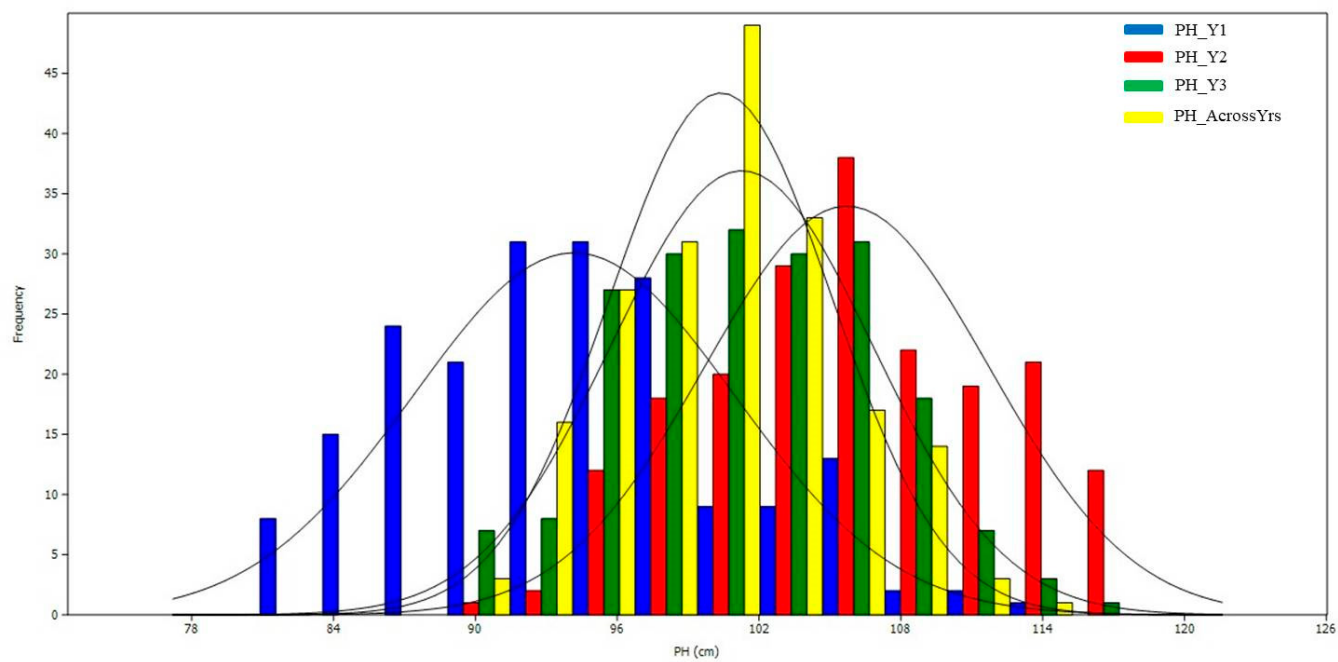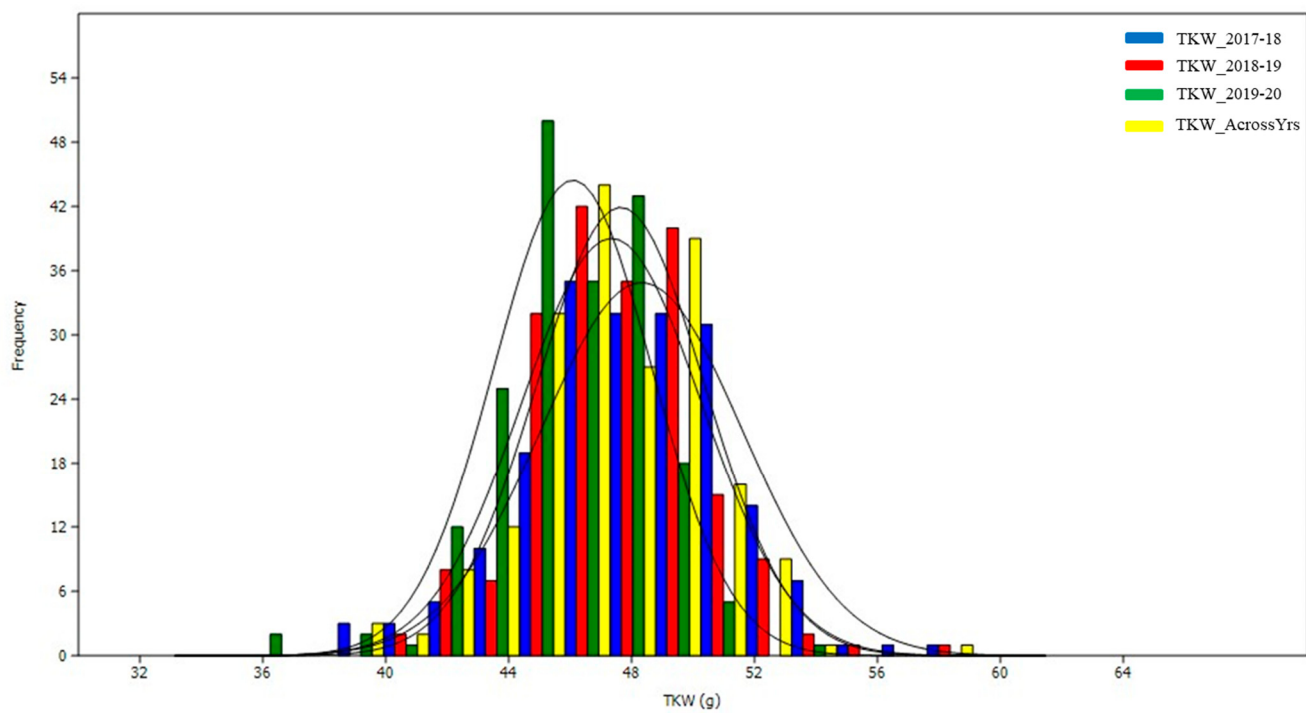

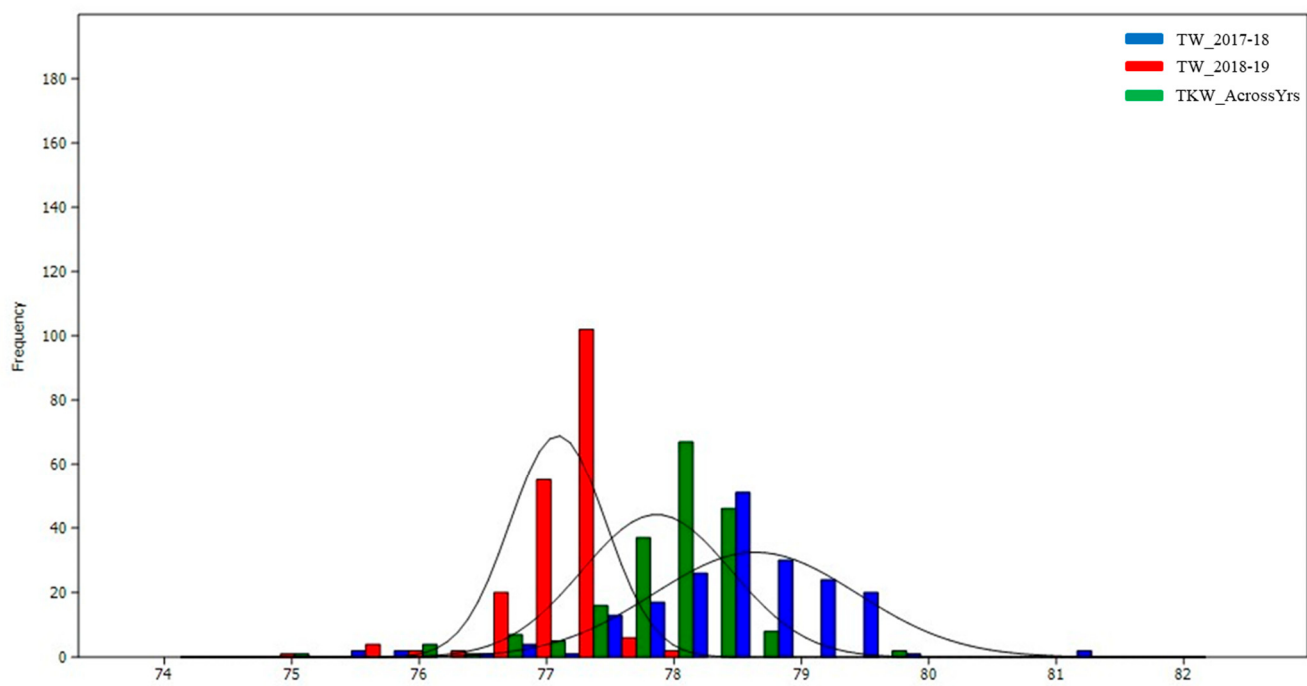

Supplement: Supplementary file 1 [file plants-12-00220-s001.zip › plants-2061784-supplementary.pdf]
